# Screening for recombinants of *Crambe abyssynica* after transformation by the pMF1 marker-free vector based on chemical selection and meristematic regeneration

**DOI:** 10.1038/srep14033

**Published:** 2015-09-11

**Authors:** Weicong Qi, Iris E. M. Tinnenbroek-Capel, Elma M. J. Salentijn, Jan G. Schaart, Jihua Cheng, Christel Denneboom, Zhao Zhang, Xiaolin Zhang, Han Zhao, Richard G. F. Visser, Bangquan Huang, Eibertus N. Van Loo, Frans A. Krens

**Affiliations:** 1 Jiangsu Key Laboratory for Bioresources of Saline Soils, Provincial Key Laboratory of Agrobiology, Institute of Biotechnology, Jiangsu Academy of Agricultural Sciences, Nanjing 210014, Jiangsu, People’s Republic of China; 2 Wageningen UR Plant Breeding, Wageningen University and Research Centre, PO Box 386, 6700 AJ Wageningen, The Netherlands; 3 Department of Ornamental Horticulture, China Agricultural University, Beijing, 100193, China; 4 College of Life Science, Hubei University, Zip code 430062, Wuhan City, China

## Abstract

The T-DNA region of pMF1 vector of marker-free system developed by Wageningen UR, has *Recombinase R-LBD* gene fusion and *nptII* and *codA* gene fusion between two recombination sites. After transformation applying dexamethasone (DEX) can activate the recombinase to remove the T-DNA fragment between recombination sites. The recombinant ought to be selected on 5-fluorocytocine (5-FC) because of codA converting 5-FC into 5-fluorouracil the toxic. A PMF1 vector was transformed into hexaploid species *Crambe abyssinica*. Two independent transformants were chosen for DEX-induced recombination and later 5-FC selection. In contrast to earlier pMF1 experiments, the strategy of stepwise selection based on meristematic regeneration was engaged. After a long period of 5-FC selection, recombinants were obtained successfully, but most of the survivors were wildtype and non-recombinant. The results revealed when applying the PMF1 marker-free system on *C. abyssinica*, 1) Increasing in the DEX concentration did not correspondingly enhance the success of recombination; 2) both of the DEX-induced recombination and 5-FC negative selection were apparently insufficient which was leading to the extremely high frequency in chimerism occurring for recombinant and non-recombinant cells in tissues; 3) the strategy of stepwise selection based on meristem tissue regeneration was crucial for successfully isolating the recombinant germplasm from the chimera.

There is a lot of controversy about genetic modification (GM) of crops, while the research on positive or negative aspects of GM crops is still going on. From scientific literature it is clear that GM crops can be beneficial for people, planet and profit with sustainable improvements of quantity or quality of plant products^1^. However, for the continuation of the GM research and the application of its products in future, wide general social approval is a prerequisite and it is unlikely that this will be achieved soon. The main problem with many people, non-governmental organizations and governments is the uncertainty about the safety of GM crops. A common argument is that the food produced from GM organisms might be potentially harmful to human health because of toxicity or allergenicity. However, this can be tested before a new GM crop is brought to the market^2^. So with a proper test system, this risk can be minimalized. However, because food is directly consumed by people, there is always a chance that they will remain sceptic about GM foods. In comparison to the food crops, GM non-food crops might have better possibilities for acceptance by the general public. Another vital point in the discussion on GM crops is concerned with the marker genes used for selecting transformation events. At present, those markers are mainly genes coding for antibiotic or herbicide resistance. There is concern about the possibility that when GM crops with antibiotic resistance genes are grown in the field, there will be a chance of horizontal gene flow of the these genes into the genomes of the microorganisms living in the soil. This might lead to the development of antibiotic resistant pathogens^3^. Similarly for herbicide resistance some people fear that by crosspollination between a GM crop and wild (weedy) relatives a kind of super weed will be created^4^.

To avoid the above-mentioned risks, it is better to produce transgenic crops without antibiotic and/or herbicide resistance genes or any other sequences that are not desired in the final product. Some novel selection strategies making use of other selective agents than herbicides and antibiotics have been developed, for example the positive selection method using the *Streptomyces rubiginosus*xyl A gene in the T-DNA^5^. These new marker genes are regarded as less risky, but because they are mostly from microbiological origin, they still run the risk of being disliked by the public. Therefore, other strategies for transformation have been developed, such as the marker-free system. Till now, several systems have become available to obtain marker-free GM crops^6^,^7^,^8^,^9^,^10^,^11^,^12^,^13^,^14^,^15^. Wageningen UR Plant Breeding developed some of these. One of them is based on marker excision and contains an *R recombinase* gene from *Zygosaccharomyces rouxii* fused to the ligand-binding domain (LBD) of the rat glucocorticoid receptor. This gene fusion is under control of a 35 S promoter^16^ that results in a continuous and ubiquitous expression of the combined gene in the transformed plant. Because cytosolic factors will bind to the LBD, the R recombinase-LBD protein complex cannot enter the nucleus. When transformed plant cells are exposed to the chemical dexamethasone (DEX), this will initiate competition for the LBD binding sites. With DEX bound to the LBD, the R recombinase-LBD protein is able to enter the nucleus. Here, it induces recombination and excision of DNA that lies between the recombination sites (RS). Gene sequences between these recombination sites, so including the marker gene, can be removed in this way. The PRI system uses an neomycin phosphotransferase *II* (*nptII)* gene^17^ still as the selectable marker, but it is fused to a cytosine deaminase gene (c*od*A)^18^ of *E. coli*, which allows negative selection against transformed cells without recombination^10^. This is done by placing transformants on a medium with non-toxic 5-fluorocytosine (5-FC). The 5-FC will be converted into the toxic compound 5-fluorouracil (5-FU) by action of the *cod*A protein part enabling selection of successfully recombined cells but eliminating those without recombination. The CaMV 35 S promoter drives the combined c*odA-nptII* gene for expression in all tissues. Both the R-recombinase-LBD gene and the *cod*A-*nptII* gene are placed between the recombination sites so that they will be removed after recombination and subsequent selection. This entire system is present in a binary vector called pMF1^19^, which is known as the marker-free system developed by Wageningen UR (http://www.wageningenur.nl/en/Expertise-Services/Research-Institutes/plant-research-international/Products-Facilities/Markerfree-technology.htm). In addition to the marker removal system between the recombination sites this vector also contains a multiple cloning site (MCS) that can be used for insertion of genes of interest, outside the recombination sites.

Previously, we have report a series methods for *C. abyssinica in vitro* regeneration and agrobacterium mediated gene transformation. Here we report applying the pMF1-based, marker-free system of WUR Plant Breeding to *Crambe abyssinica* (crambe) genetic modification with these methods. Crambe is a non-food oil seed crop^20^,^21^,^22^. Its seed oil has a wide range of potential applications in chemical industry because of the high erucic acid content^23^,^24^,^25^. Furthermore, it is also a potential platform crop for various other kinds of feedstock oil for industry using genetic modification^26^,^27^. Hence, producing marker-free crambe is considered to be a prerequisite with respect to increasing consumer acceptance and to allowing retransformation for further improvements if required. In the research presented here, a model construct, pJS-M14, derived from the pMF1 marker-free system, was used carrying two reporter genes to monitor individual steps in the process of transformation of the non-food oil seed crop crambe. For this new and potential industrial crop, a novel way to provide the DEX treatment and 5-FC selection combined with the regeneration system based on explants with meristematic tissues lead to the development of a new method for the production of marker-free plants, still using induction of recombination. In contrast to earlier pMF1 experiments^10^,^12^ on other crop or plant, the strategy of stepwise selection based on tissue regeneration engaged here was pronounced and particularly suited for crambe. Summarily, here we showed how tissue regeneration efficiently facilitated an inefficient plant recombination system to give the wanted recombinant.

## Results

### Determination of the effect of dexamethasone on regeneration

Axillary buds from *in vitro* grown wild type (WT) plants were subjected to regeneration medium with various DEX concentrations (0, 5, 15, 25 μM). After 4 weeks, regeneration frequencies were scored. All explants treated with DEX, irrespective of the concentration used, showed no differences in visual appearance with respect to bleaching or necrosis and showed similar regeneration frequencies as the ones without DEX treatment. In all cases, the percentage of explants giving regeneration was around 95%. So, there were no indications for a significant effect of the *in vitro* DEX treatment on the regeneration of shoots from WT axillary buds. The same DEX concentrations were used later in the DEX treatment given to the pJS-M14 transgenic plant material to induce excision.

### Determination of the proper concentration of 5-FC for selection

Application of 5-FC in the regeneration medium at any of the concentrations (0, 10, 50, 100 and 500 mg·L^−1^) tested did not show any significant effect (positive or negative) on the regeneration from axillary bud explants in 4 weeks (data not shown).

As an effect of 5-FU, the toxic derivative of 5-FC after conversion by action of cytosine deaminase (CodA), regenerating shoots from the axillary bud explants turned white (the bleaching started from the shoot tip and then to the bottom), while those on medium without 5-FU stayed green. The treatment of axillary bud explants with 5-FU in regeneration medium at concentrations of 50 mg·L^−1^ and 100 mg·L^−1^ showed complete bleaching in all subjected WT explants after 4 weeks. The 5 mg·L^−1^ 5-FU treatment on axillary bud explants showed no visible effect, the regeneration of the explants and the colour of the regenerating shoots were the same as that of the 0 mg·L^−1^ treatment. For the treatment with 10 mg·L^−1^ 5-FU, only two explants were found with some bleaching in regenerating shoots, which also indicated insufficient selection. The data are presented in Online Resource 1.According to the results of 5-FC and 5-FU, in later selection for recombinant, 200 mg·L^−1^ 5-FC was used in all experiments. This, assuming that a conversion rate of only 25% of 5-FC into 5-FU would already be enough to allow efficient selection.

### Transformation of crambe with the PMF1 vector pJS-M14

**Figure 1:**
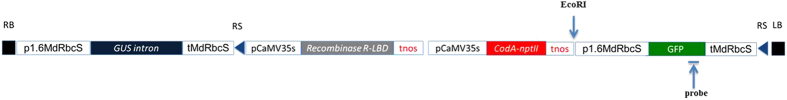
T-DNA organization of the binary plasmid used, pJS-M14. RB is right border; LB is left border; there are two recombination-sites (RS), and in between them there are 3 genes (combinations), i.e. *Recombinase R-LBD*, c*odA-nptII* and *gfp*. Outside the RS sites there is the marker gene *gusintron*, acting as gene-of-interest. The *gusintron* and *gfp* were both driven by apple 1.6 kb Rubisco promoter and apple Rubisco terminator (Schaart *et al.*, 2011). After recombination, the genes between the RS sites will be removed, while the *gusintron*gene will remain. The unique restriction site *Eco*RI is used for digestion prior to Southern blotting; the*gfp* gene is the target for probing.

Binary vector pJS-M14 (Fig. 1) contains the *gfp* gene as reporter for successful transformation and excision (present: no excision yet; absent: excision) and the *gus* gene representing gene-of-interest, meant to stay behind after excision. From 400 inoculated explants, multiple green regenerating shoots were obtained after 20-weeks of Km selection. Sixteen independent transformation events were isolated, and GUS staining and PCR analysis proved their transgenic nature. The fluorescence of the *gfp* controlled by apple 1.6 kb Rubisco promoter were detectable only in etiolated seedling of the transgene crambe, but not in any other kind of plant or tissue.

**Figure 2:**
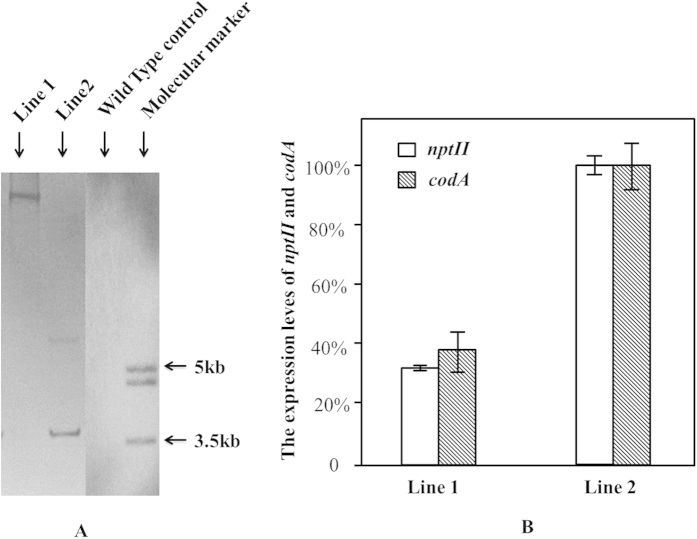
Southern blot and qPCR analysis of the selected T0 lines. To evaluate the t-DNA insertion number in T0 plants of Line 1 and Line 2, Southern blotting analysis was conducted on the pooled genomes DNA sample of T1 progeny plants of them respectively, as showed in (**Chart A)** with WT as control. The outer right lane shows a molecular weight marker. Hybridizing fragments should have a minimal size of 2.8 kb. The (**Chart B**) provides the qRT-PCR data on the expression of the *nptII* gene and the *codA* gene in the *in vitro* leaf material of the two selected original T0 plants without any treatment. The average relative expression level of the highest performing line (Line 2 for both genes) was set at 100%. Statistical analysis doing a T-test (student t-test) showed that the difference in expression between both lines was significant (p < 0.01) for both genes.

A T0 line with single T-DNA insertion (Line 1) and another one with double T-DNA insertion (Line 2) were chosen for triggering recombination by DEX treatment. The T-DNA insertion number of these T0 plants was evaluated by the southern blotting conducted on the pooled genome-DNA-samples of T1 progeny plants (Fig. 2A). A qRT-PCR analysis on the expression levels of the *npt*II and *cod*A genes in T0 plants indicated that the introduced genes were indeed expressed in both lines but had a significantly stronger expression in the Line 2 than in Line 1 (Fig. 2B).

After being chosen, these two independent transformants were amplified by the method of axillary bud regeneration. And then the multiplied regeneration shoots were given DEX and 5-FC treatments stepwise, as described in ‘material and method’ and the Table 1.

### The effect of theDEX treatment on rooting of *in vitro* shoots

**Figure 3:**
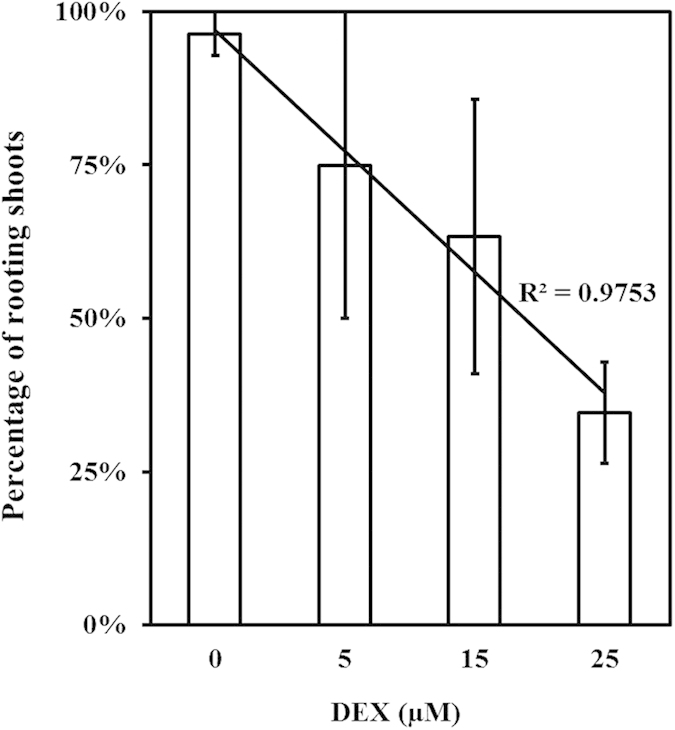
The effect of DEX treatment on rooting of *in vitro* shoots. The effect of a 6-week DEX treatment on the rooting of shoots is demonstrated. The percentages of shoots giving roots on media with different DEX concentrations are given, together with the standard error of means as bars on the columns. The percentage of rooting shoots was significantly correlated (at 0.05 level) with the DEX concentration according to Pearson correlation analysis (2-tailed) in SPSS.

The different DEX treatments were administered to regenerating shoots *in vitro* through the rooting medium. Although in previous experiments, no effect of the DEX on shoot regeneration from WT axillary buds was found, here, high concentrations of DEX (Table 2), unexpectedly, did show a negative effect on the rooting of the inoculated transgenic shoots. As shown in Fig. 3, DEX concentrations of 15 and 25 μM gave lower rooting percentages. The negative correlation between rooting and the DEX concentration was found to be significant by correlation analysis following Pearson (2-tailed).

### The efficiency of recombinant plant generation as monitored by the treatments with 5-FC and Km at Step 4

**Figure 4:**
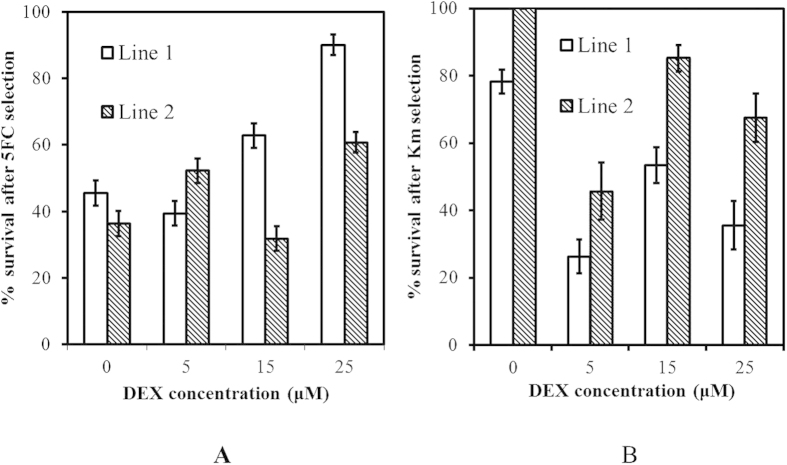
The effects of 5-FC and kanamycin on survival of *in vitro* shoots. The effect on survival of regenerating shoot clusters of 5-FC and Km at Step 4 is shown. Panel A demonstrates the effect of 5-FC selection for both lines after different treatment with DEX; Panel B gives the effect of Km selection. The bars on the column represent the standard error (SE).

As shown in Table 1, the regenerating shoots in Step 4 from the axillary bud explants from transgenic crambe lines were cut and subjected to cultivation on either 5-FC or Km for 3 weeks. During this selection period, the individual shoots kept regenerating and became regeneration clusters at the end of the term. The regeneration clusters consisted of green shoots, white shoots or a mixture. The survival rate is defined as the number of clusters that still had green shoots left. The aim was to study whether there was a concentration effect of DEX on the excision efficiency. The survival rates after the 5-FC selection for 3 weeks as an indication for successful excision are presented in Fig. 4A. According to a Chi-square test, the survival rates of DEX (5, 15, 25 μM) treated material of both lines were significantly higher than the ones without DEX. Comparing the two GM lines, their 5-FC survival rates were significantly different from each other, with the fraction of 5-FC survival being generally lower for line 2 than for line 1. Moreover, the survival rates after Km treatment, as an indication for no excision, also showed differences related to the various DEX treatments. Figure 4B displays the rates of subjected explants giving no bleaching of shoots. Surprisingly, the explants of line 1 without DEX already showed bleaching of regenerating shoots at 12.8%, while the materials without DEX from line 2 showed no bleached shoot at all, as expected. Chi-square tests also showed that on Km selection, the shoot-clusters on any treatment with DEX (5, 15, 25 μM) gave significantly lower percentages of surviving shoots than the explants on 0 DEX; no significant differences were found between the various concentrations tested. The material of line 1 gave more serious bleaching than line 2.

### Survival of regenerating shoot-clusters after each step of 5-FC selection

**Figure 5:**
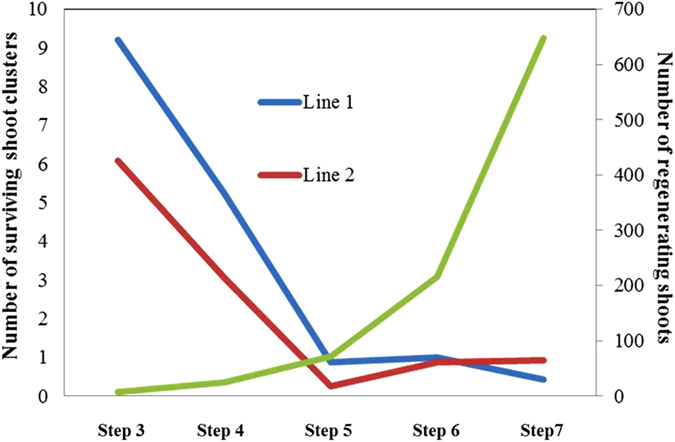
The efficiency of 5-FC selection. The efficiency of 5-FC selection is demonstrated by plotting survival at different step, 3 to 7 against the theoretical multiplication rate that can be achieved without any selection. Blue and red lines (with Y axis on the left) display the dynamics of shoot survival for respectively line 1 and line 2. The start value was calculated as the total number of shoots starting 5-FC selection, from the axillary bud regeneration, divided by the number of initial rooting plants times three as the number of axillary buds isolated from them. The start number for line 1 is nine, and for line 2 is six. As start value for the ideal curve eight was taken which is close to the average start-value of line1 and line 2. The curve in green (with Y axis on the right) shows the ideal shoot multiplication curve starting from eight shoot in the beginning, assuming that one shoot in regeneration medium for 3 weeks without selection will produce 3 shoot clusters on average. Then, after 4 rounds of shoot to shoot amplification, there will be 648 shoots finally.

The regeneration medium was used as the basic medium for the selection in each step. So, there was always regeneration in parallel with selection. As showed in Fig. 5, According to the results of preliminary experiments without selection, each shoot subjected to regeneration medium for 3 weeks would give rise to at least 3 newly regenerating shoots leading theoretically to 243 shoots after four rounds of multiplication; comparing this number with the actual number of regenerating shoots as obtained after treatment with DEX and selection on 5-FC,it is clear that as a result of the 5-FC selection, the number of surviving shoots was constantly decreasing in every step. The actual numbers of surviving shoot-clusters at the end of each step are shown in the Table 3. At the end of Step 7, there were 18 surviving shoots for line 1, and those were obtained from 8 regenerating axillary buds of Step 2, and from 5 originally rooting plants in Step 1; for line 2, there were 15 survivors, which were derived from 4 axillary buds of Step 2, and from 4 rooting plants of Step 1(Table 3).

### GUS staining of surviving shoot-clusters after prolonged 5-FC selection

**Figure 6:**
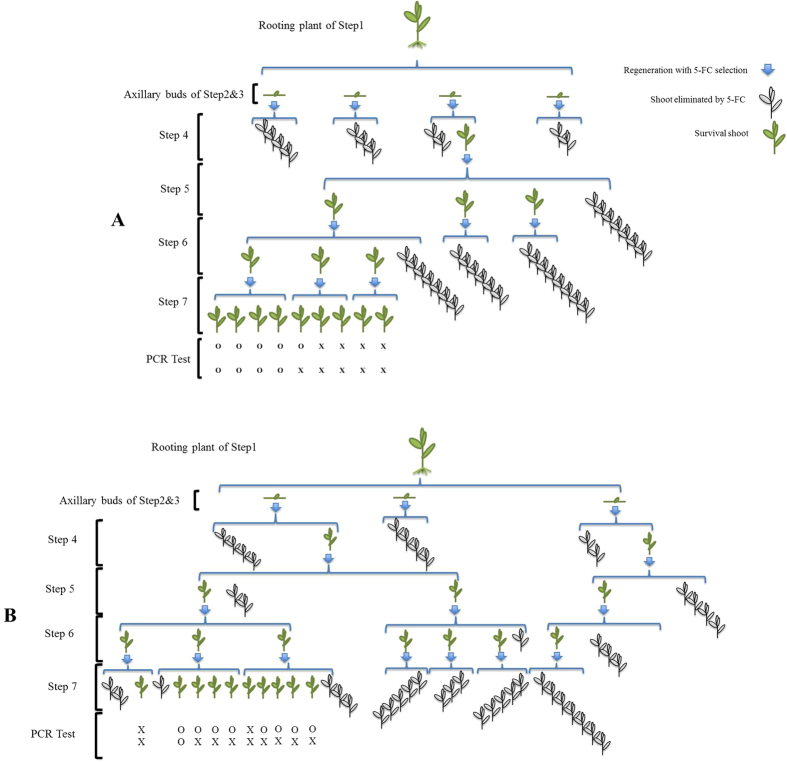
Flow chart representation of the steps taken to come to a maker-free crambe GM plant. The (**chart A,B**) exemplifies the strategy engaged in this approach to obtain individual recombinant shoots from a two rooting plants (Step1) of line 2 with the 15 μM DEX treatment. There were 5 axillary buds obtained from the shoot in total, and 4 from A and 1 from B. As showed in the right corner, the blue arrows mean a round of regeneration and selection with 5-FC; plants in pale represent those killed by the selection, and green plants indicate the survivors. The drawn numbers are the actual number of plants handled. Gus-staining at the end of Step 6 and 7 were all positive for the green individuals. The ‘PCR Test’ showes the results of two PCR analyses on the surviving shoots above. ‘O’ means recombinant, and ‘X’ means non recombinant. So, finally from this specific starting plant, 9 surviving shoots were obtained and within them, there were 4 double-confirmed recombinantindivial shoots, 1 single-confirmed recombinant shoots and 4 non-recombinantshoots. Seeds from two of these double-confirmed recombinantshoots were germinated to establish seedlings for further PCR, gfp fluorescence and GUS staining analysis (Table 4) followed by performance studies on kanamycin containing media. And the chart B showed the flow-diagram of the other one recombinant regeneration shoot originated from another single rooting plant of line 2 with 15 μM DEX treatment as well.

GUS staining was done on the surviving shoots at the end of Step 6 and 7. If the shoots were GUS positive and PCR negative, they were considered as potential recombinant; in case the shoots showed positive for both tests, they were regarded as non-recombinant; if negative for both, it was considered to be wild type. Among the survivors, GUS negative shoots (as white as WT material) were found in both lines, which implied that the starting material was chimeric, containing both transformed and untransformed cells in both cases. For line 1, 14 out of 18 total green shoot clusters showed no GUS staining (without any blue color) coming from three of the five original rooted shoots. For line 2,only one of the shoot clusters proved to be GUS negative derived from one original rooted plant, while the other 14 clusters stained positive. The flow-diagram demonstrating how the recombinants were finally acquired was showed in the Fig. 6. All in all, the percentage of GUS positive shoots after prolonged selection on 5-FC, indicating putative transgenic, recombinant material, ranged from 22% (line 1) to 93% (line 2; Table 3). The GUS positive survivors of line1 originated from two Step-1 rooting plants of the 15 μM DEX treatment; those of line 2 were from three Step-1 rooting plants, one of the 0 μM DEX (spontaneous recombination) and two of the 15 μM DEX treatment.

### Identification of true recombinant plant material

PCR analysis was performed on two separate leaves from each shoot culture at the end of Step 6 and at the end of Step 7 in parallel with the GUS staining. The results showed that the surviving shoot clusters consisted of recombinant, non- recombinant or WT material or a mixture of any of the three types. Recombinant shoots identified by a negative PCR only presented a small fraction of the total number of GUS+ plants. The PCR results at the end of Step 7 showed that from the GUS positive regenerating shoot clusters, 37.5% were recombinant(PCR-) and 62.5% were non- recombinant(PCR+). All of those recombinant shoots were derived from line 2, and amounted up to 15 in total. Among them, one was from obtained from the 0 μM DEX treatment, the rest from 15 μM DEX treatment.

The putative recombinant shoots after Step 7 were evaluated again by taking another two leaves from the cluster for a new DNA isolation and PCR run, as well as for a GUS assay. The GUS staining result was positive for all and proved the transgenic nature of this material to be consistent. However, the second PCR test showed that this time among the shoot clusters, previously found to be *nptII* negative for both leaves, only 30% could be reconfirmed as recombinant, which implied that among the surviving shoots, many of them were still chimeras of recombinant and non-recombinant cells. All of the double confirmed recombinant shoots were from line 2, 15 μM DEX treatments. Ultimately, five putative recombinant candidates (reconfirmed) were obtained from two original rooting plants, four originated from one rooting plant in the beginning, and one was from another. Figure 6 provides the flow-diagram demonstrating how the recombinant shoots were finally acquired at the end of Step 7 starting from the rooting plants as the result of the strategy used in the present research.

**Figure 7:**
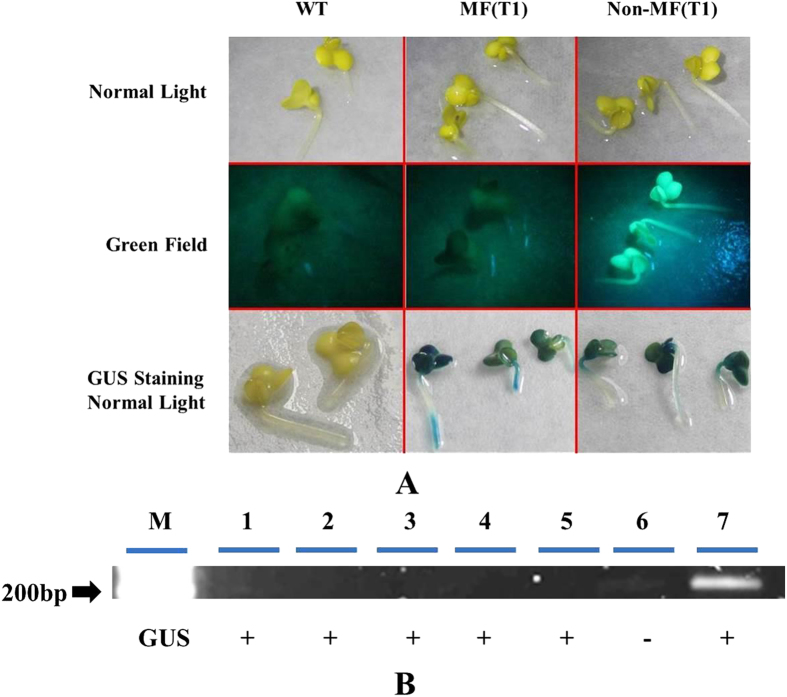
T1 seedlings tested for the absence of the T-DNA fragment in between the recombination sites. T1 seedlings originating from one of the Line 2, 15 μM DEX-treated recombinant were used for the tests showed in this figure. WT is the wild type control; Non- recombinant (T1) shows the T1 seedlings from the line 2T0 plant without DEX and 5-FC treatment. The teste for the presence of the visual markers, GUS and GFP were showed in (**Chart A**). All of seedlings shown were etiolated because that they come from seeds germinated and grown in the dark. The PCR test for the absence of the T-DNA fragment in between the recombination sites was showed in (**Chart B**). For the PCR the forward primer was located in between the recombination sites the reverse was outside. The length of amplification product is 252 bp. The number showed the lanes, 1 to 5 were the recombinants, 6 was WT, and 7 was Non- recombinant T1. The +/− (+: positive; –: negative) underneath were the results of GUS staining on the same plants.

**Figure 8:**
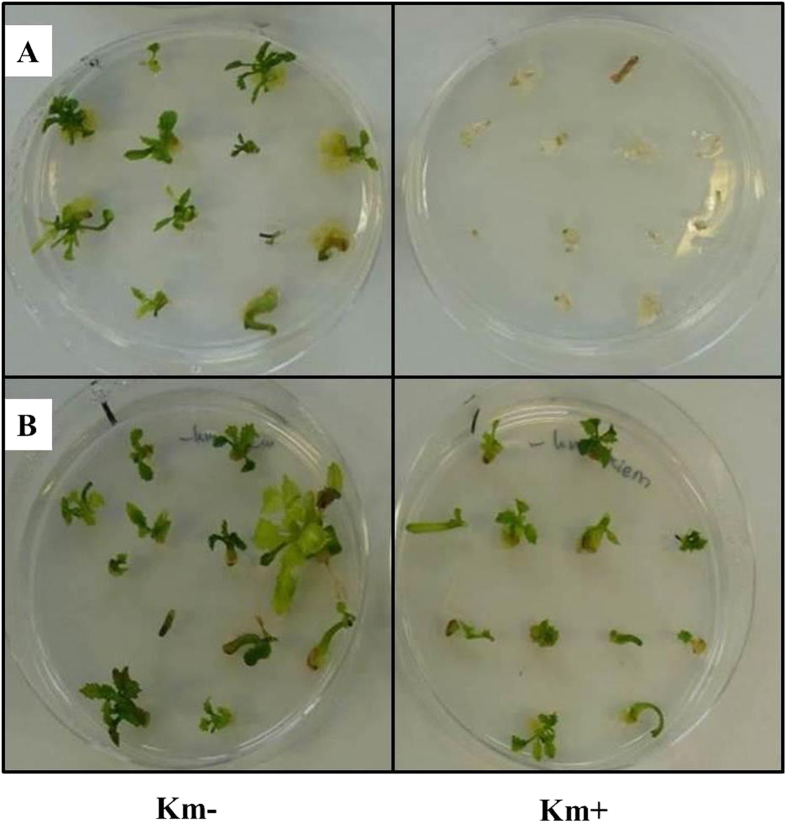
Phenotypes of the explants of recombinant exposed to kanamycin treatment. The general appearance and regeneration response is shown for cotyledonary node explants (CNE) of T2 seedlings. Two CNEs can be obtained from one seedling. Here, one is placed on medium with kanamycin (Km+) and the other one from the same seedling on medium without (Km–) as control. The orientation of the two dishes is the same. The Petri dish on the left contains no kanamycin (control); the one on the right contains 15 mg/L kanamycin. Panel A shows explants from a MF line (after excision sensitive to kanamycin) and Panel B shows a line without treated with DEX, so not recombinant.

After double negative PCR identification, all of the recombinant shoots were put onto rooting medium, and after they formed roots, they were transferred into soil and brought to the greenhouse to get rid of any remaining chimerism by going through a seed phase. As shown in Table 4, the recombinant nature was reconfirmed in the next seedling generation as proven by PCR (absence of *gfp* and *nptII*; controls being positive), gfpfluorescence^28^ and GUS staining (presence still of *gus)*. Two T1 seed families originating from two of the five putative recombinant shoots, earlier identified by PCR, were chosen for confirming their recombinant nature, using T1 seeds from a line-2 plant without DEX treatment as control. For the GUS staining, the two recombinant T1 seed families (20 seedlings tested) acted similarly as seedlings from line 2 without DEX. However, in the PCR test for the presence of *nptII* and *gfp*, the recombinant families proved to be all negative, while the original line 2 seedlings tested positive in most seedlings. Moreover, positive gfp fluorescence was observed in the seedlings originating from the line-2 plant without DEX treatment, while the seedlings of the recombinant candidates were without fluorescence, but positive for GUS staining (Fig. 7A). And PCR test also indicated the absence of the T-DNA fragment in between the recombination sites (Fig. 7B). Performance of split cotyledonary-node regenerants of the same T2 seed families on medium with or without kanamycin confirmed the homogeneous recombinant nature of the parental T1 line (Fig. 8). All explants were sensitive to kanamycin and stained blue in the GUS assay (data not shown).

## Discussion

Here we showed that it is possible to produce marker-free transgenic crambe plants using the pMF1 marker-free system from Wageningen UR Plant Breeding. In comparison with other system which can generate marker-free or cis-gene plant, the advantages of pMF1 are that 1) its recombination needs to be triggered by chemical which means increasing the chemical concentration or time of exposure may enhance the success of recombination action; 2) the pMF1 construct can be used as same as the other binary vector, unless there is exogenous applying DEX. The same mechanism has been successfully used for making marker-free crops like potatoes (slightly different from the pMF1)^29^, strawberries^10^ apple^12^ and pear^30^ before. Although the pMF1 system is supposed to work generally in plant species, the reality is that it only succeeded in strawberries, apple and pear. In those reported experiments of pMF1 generally leaf explants were taken and submerged overnight in liquid medium with DEX (10 to 50 μM). In the next step, the explants were put on regeneration medium with DEX at a concentration of 1 μM still being present and supplemented with 150–250 mg·L^−1^ 5-FC for selection for approximately four weeks. Regeneration shoots of recombinant were obtained from these explants using this protocol. In preliminary tests, we have also performed the same protocol of submerging explants on crambe aiming to acquire recombinant plantlets, but failed. No recombinant could be obtained in this way (results not shown). Therefore, we tested application of the DEX treatment to intact crambe *in vitro* shoots at rooting phase, allowing uptake of DEX through the roots. This method was used earlier in Arabidopsis^31^. In our new protocol, the treatment with DEX is continued into the next phase where axillary bud explants are isolated from the rooted shoots and put on regeneration medium. The total exposure period of the plant material to DEX is 10 weeks with 5-FC selection initiated in the last two weeks in addition to the DEX treatment. Comparing the present method with the one applied to strawberry, the DEX concentration is higher and the 5-FC selection was longer and more stringent. The most significant difference was the stepwise selection strategy aiming to enrich for recombinant cells and shoots, and to get rid of the non-recombinant cells. In the end, albeit at low frequency and long term, presentpractice was successful in acquiring PMF1recombinant in crambe.

**Figure 9:**
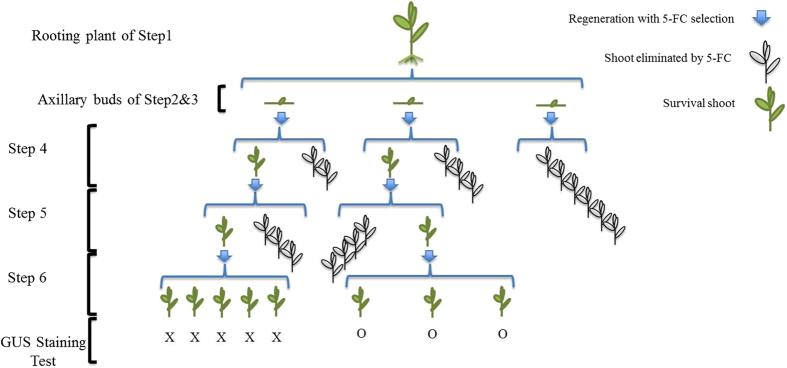
The process in which wild-type vegetation tissues were identified from a chimeric crambe GM shoot. This figure exemplifies the process in which wild-type vegetation tissues were identified from a chimeric shoot of line 2 after the 15 μM DEX treatment and 5-FC selection. There were 3 axillary buds obtained from the shoot in total. As showed in the right corner, the blue arrows mean a round of regeneration and selection with 5-FC; plants in pale represent those killed by the selection, and green plants indicate survivors. The drawn numbers are the actual number of plants handled. All the survivors were tested by Gus-staining at the end of Step 6, ‘O’ means GUS positive, and ‘X’ means negative. So, finally from this specific starting plant, five surviving shoots obtained were GUS-negative, and three were GUS-positive. And this result gives a thorough indication of the chimeric nature in the starting shoots.

In Fig. 9,it is a flow-diagram demonstrating how the WT shoots werefinally isolated. The recovery after 5-FC selection, of such a high percentage of WT non-transformed shoot clusters demonstrated that the transformation protocol developed in our lab for crambe using cotyledonary node explants (CNE) with meristematic tissue^32^ may give rise to chimeras. However, such chimerism was never observed in earlier or later transformation experiments when looking e.g. at segregation ratios in T1 or T2 progenies of transgenic lines. A possible reason can be that here a less stringent (shorter) Km selection was applied and the continued 5-FC selection without Km allowed the scarce WT cells to proliferate and become prominent. Still, it was obvious that in the experiments described here chimerism did occur in the original shoots put on rooting medium and subjected to DEX; line 1 showing a higher percentage of WT cells (2.4% of the original number of axillary buds contained WT cells) than line 2 (0.9%). The two lines used for establishing the protocol were analyzed at the start by GUS staining and molecular analysis by PCR and Southern hybridization and proven to be transgenic. The two lines were selected based on their difference in T-DNA copy number with line 1 having a single insert and line 2 having more than one insert to monitor any differences in efficiency in obtaining recombinant plants. In crambe, transformation usually gives 49% single insert and 35% 2–3 copy inserts^32^. The PCR and Southern hybridizations apparently cannot exclude the possibility of a few WT cells to be still present. There was no evidence indicating that any axillary-bud explant from the rooting shoots in the beginning was totally non-transgenic (WT). Using the CNE protocol of transformation in crambe, a stringent and prolonged selection period on Km seemed to be required in order to avoid the survival of WT cells^32^. Final assurance for obtaining fully transformed plants is to go through a seed phase and working with T1 or T2 generations. Moreover in present research, a passage through a seed-phase should also ensure the homogeneous recombinant nature of the subsequent generations.

Using a multicellular organ or tissue to treat with DEX also allows for the generation of random mosaic chimeras existing of recombinant and non-recombinant cells. If not followed by a regeneration protocol based on the outgrowth of shoots from one cell (adventitious shoot formation) the plant material will remain chimeric. This was what was found here, because in crambe the regeneration process is based on axillary bud explants carrying meristematic tissue and shoots will originate from multiple cells. A stringent selection scheme is required to eliminate non-MF cells and although it was clear that in our experiments 5-FC selection really helped in reducing regeneration of non-MF cells, it did not totally prevent it. Although 150 mg/l 5-FC was reported to be sufficient in other crops^10^,^12^, for crambe a higher concentration than the 200 mg/l we used might lead to better results. For example, at the end of Step 7, the surviving shoot clusters were very likely still chimeras mostly, as clear from the two separate PCR tests performed on DNA samples taken from different individual leaves. The results clearly showed that both the DEX treatment and the 5-FC selection did work but not to a full 100%. In our experiments we could not find an optimal DEX concentration when checking survival on Km and 5-FC at step 4. For line 1 the survival on 5-FC suggested that 25 μM was best but this was not verified by survival or rather bleaching on Km. For line 2, 15 μM gave the lowest level of survival on 5-FC and the highest survival on Km, suggesting poor performance for generating recombinant plants. However in the end, most MF shoots were obtained from this particular treatment. The effect of Km was expected to be opposite to the effect of 5-FC. Such a straightforward negative correlation was not found. Statistical analysis taking all DEX treatments together and comparing to non-treated controls showed that there was a significant difference between with or without DEX treatment. As mentioned earlier, most recombinant candidates were isolated from shoots and explants treated with 15 μM DEX, so for further research this concentration can be recommended. The one recombinant line obtained from the control treatment without any DEX can be explained by spontaneous recombination; a phenomenon that was observed earlier in lily (Krens pers. commun.) and could come from the Recombinase–LBD fusion protein being able to pass the nuclear membrane without DEX attached to it nor any other proteins.

No recombinant shoots were found for line 1, the line with the one copy T-DNA insert where recombination should result in a simple one copy remaining T-DNA with the *gus* gene as single gene-of-interest left between the T-DNA borders. A possible explanation for this could be the generally low efficiencies of the DEX treatment and the subsequent 5-FC selection in this system and the presence of a relatively large portion of non-transgenic cells competing with the recombinant cells for growth and regeneration on 5-FC medium. The position effect of the T-DNA insertion^33^ might play a role in determining accessibility of the Rs sites in the T-DNA to the Recombinase. Moreover, we demonstrated that the expression of the introduced genes (Fig. 4) was higher in line 2 carrying two copies of the T-DNA than it was in line 1 (one copy insert. The higher expression level of the exogenous genes in line 2 could have resulted in a higher excision rate and could explain the more efficient recovery of recombinant plants in that line. Therefore, the results implied that for making marker-free plants with the pMF1 system, the nature of the original transgenic material is vital for the final success and multiple independent transgenic lines should always be taken. The experimental procedure and result revealed interesting points which was not reported in any published researches. 1) In the DEX treated transformed Crambe explant, the chemical triggered T-DNA recombination succeeded only in partial somatic cells, but failed in others. 2) The 5-FC chemical negative selection was insufficient to eliminate those non-recombinants. For these two reasons, it led to heterogeneity among somatic cells, which means the chimerism. 3) Stepwise tissue-regeneration and selection based on meristematic regeneration was pronounced for isolating the wanted germplasm from the chimera. The result also demonstrated that with the strategy, 1) PMF1 could be applied on other plant like crambe, for instance rapeseed and *Camelina sativa*; 2) the potential of this vector system to produce, in the future, a marker-free GM crambe crop with altered traits, e.g. in oil composition that might be more acceptable to the general public.

The oil composition is the core value of crambe. And there have been some genetic modification experiments targeting on its seed oil biosynthesis to improve its seed oil quality^34^,^35^, which indicated the potent of oil metabolism manipulation. Oil metabolism in plant seed is a network. Mostly manipulating seed oil composition needs the regulation on multiple genes. For example, to improve crambe erucic acid content in its seed oil, RNAi of endogenous *fatty acid desaturase 2* (*CaFAD2-RNAi*), over expression of exogenous *fatty acid elongase* from *Brassica napus* (*BnFAE*), and over expression of exogenous lysophosphatidic acyltransferase from *Limnanthes douglasii* (*LdLPAT*) were necessary^35^. Beside erucic acid, crambe has been considered to become a bio-platform to produce high value chemical compound like Jojoba wax ester, which is in need of enhancing *FAE*, suppression on endogenous *FAD2*, Jojoba *Fatty acid reductase* and *wax synthase*^27^. To achieve the goals, marker-free genetic modification (MF-GM) is always an attractive alternative. It is interesting to build a single-gene MF-GM crambe library, in which each of the lines will only carry one transgene involved in oil metabolism. (The performance of the transgene in each line should be strong and stable, for which homozygous GM lines will be preferred.) So this library will consist of many different MF-GM crambe lines with various transgene germplasm, while the establishment of the library will greatly facilitate the transgenic research or breeding on crambe seed oil. For instance when aiming at high erucic, low polyunsaturated fatty acids or Jojoba wax ester, researchers will only need to do crossing between different GM lines from the library until all the genetic modification natures (as described above) wanted are assembled into one single plant. And the marker-free system will also allow retransformation with the same selection marker constantly. For the convenience of verifying the successful hybrid, it is better to link the transgenes with different visible markers, e.g. GFP, RFP and GUS.

## Material and Methods

### Plant material, vector and transformation

Seeds of crambe cv. ‘Galactica’ were sterilized and germinated on medium (full MS, 20 g·L^−1^ sucrose + 8 g·L^−1^Phytoblend; pH 5.8) for 7 days to obtain cotyledonary node explants as starting material for transformation and regeneration^32^. The growth chamber was set at a photoperiod of 16 h with a light intensity of 4000 Lux and a temperature of 24 °C. The transformation was carried out as described in Online Resource 1.Binary vector pJS-M14 (Fig. 1) harboured by *Agrobacterium tumefaciens*strain AGL0^32^ was used in this study.

### Verification of transformation

Histochemical GUS staining was carried out as described by Jefferson^36^.

To evaluate the t-DNA insertion number in T0 plants of Line 1 and Line 2, Southern blotting analysis was conducted on the pooled genomes DNA sample of T1 progeny plants of them respectively, with WT as control. Twenty T1 progeny seedlings of 20-day after germination were pooled together for genomic DNA isolation both in Line 1 and Line 2 respectively. The DNA isolation method was as same as described Aldrich and Cullis^37^ but with 1% (w/v) polyvinylpyrrolidone-10 in the DNA extraction buffer. Probe design and restriction enzyme selection were based on the sequence of *gfp*. The location of the probe (566 bp) is given in Fig. 1, as well as the restriction sites of *Eco*RI which was used to digest the DNA. The digested DNA samples were fractionated on a 0.8% (w/v) agarose gel and transferred to Hybond N+ membrane (Amersham Biosciences, UK) according to the manufacturer’s recommendations. The labelling system was the DIG-High Prime DNA Labelling and Detection Starter Kit I, Roche (Cat. # 11745832910), and conducted according to its introduction manual.

Primers, specific for the *nptII* gene and *codA* gene respectively, were developed for qRT-PCR based on their sequences. Total RNA was extracted from 0.5 g leaf material of *in vitro* plant with RNeasy Plant Mini Kits (Qiagen, Germany). The isolated RNA was treated with RNase-free TURBO DNase (Ambion, USA), and then first-strand cDNA was synthesized in 20 μl from 1 μg of total RNA with iScript™ cDNA Synthesis Kit (Bio-rad, USA). PCR reactions were performed in triplicate. The expression of each replicate was normalized by the reference gene, β-actin 2 34,38,39. The relative expression level of each replicate was calculated according to the comparative Ct method (User bulletin no. 2, ABI PRISM 7700 Sequence Detection System, December 1997; Perkin-Elmer, Applied Biosystems).

The information about the primers and cycling conditions used in the present research is given in Online Resource2.

### Determination of the effect of dexamethasone on regeneration

To test the influence of the DEX treatment on regeneration of *C. abyssinica* explants, axillary bud explants were cut from rooted, *in vitro* grown plants and placed on regeneration media containing various concentrations of DEX (0, 5, 15 and 25 μM). After 4 weeks, the phenotypes of the explants and regenerating shoots were monitored compared to the controls (0 DEX) and their regeneration responses were scored as percentage of explants showing regeneration of the original number of explants exposed to the treatment.

### Determination of the effect of 5-FC and 5-FU on regeneration

Similarly as in the previous paragraph, axillary bud explants were taken and transferred to regeneration media, this time containing 5-FC in concentrations of 0, 10, 50, 100 and 500 mg·L^−1^ or 5-FU in the concentrations of 0, 5, 10, 50, 100 mg·L^−1^. After 4 weeks, phenotypes and regeneration responses of these explants were scored as described above.

### Application of DEX and subsequent selection steps

The general setup of this experiment with its individual treatments is displayed in Table 1.

### DEX treatment to induce recombination (Step 1, 2 and 3)

Two independent T0 lines were chosen, of which one has a single T-DNA insertion (line 1), the other has a double insertion (line 2). The two T0 lines were vegetatively propagated by cutting axillary buds from *in vitro* grown plantlets to have enough regenerating shoots for the DEX treatment. The DEX treatment was applied through incorporation in the rooting medium on which multiplied shoots were placed. The DEX concentrations tested were 0, 5, 15 and 25 μM. Table 2 shows the number of shoots from each line put on rooting medium for each DEX concentration (each original shoot was marked with a unique number); the shoots were subjected to DEX through the rooting medium for six weeks; after that, axillary buds (the numbers are given in Table 2) from these rooted shoots were cut and put for two weeks onto regeneration medium with the same DEX-concentration as the rooting medium they were from. After these two weeks of DEX treatment of axillary bud explants, the regeneration medium was renewed, and the new medium was supplemented with 200 mg·L^−1^ 5-FC as selectable agent together with DEX for another two weeks (each axillary bud had a unique code to mark its origin).

### Selection for recombinant shoots (Step 4 to step 8)

In general, regenerating shoots from the axillary-bud explants were isolated and taken for further selection. The regeneration medium was used as basic medium in all subsequent passages. During every new culture cycle, the isolated shoots grew into regeneration clusters again. The duration of every culture cycle was three weeks. At the end of each period, the condition of these clusters was recorded; they were either entirely green, or entirely white (bleached) or a mixture. Only healthy, green shoots were taken for the next passage. After Step 3, the regenerating shoots were divided into two groups evenly, one was for 5-FC (200 mg·L^−1^) selection (continued, negative selection), and the other for Km (10 mg·L^−1^) selection in order to get an idea about the efficiency of the DEX treatment. After 3 weeks the condition of all transferred shoots was monitored. At the end of Step 4, the regeneration clusters on 5-FC selection were split into individual shoots and the healthy ones were kept for the next 5-FC selection step. The clusters from Km selection, however, were discarded after their condition was recorded. In Steps5 to 8, only 5-FC selection was applied at 200 mg·L^−1^.

At the end of Steps 6 and 7, after the clusters were split up into individual shoots, tissue from every shoot was taken for GUS staining. In addition, some of these shoots were tested by PCR. The shoots for PCR at the end of Step 6 were randomly selected (all the clusters were covered), but the shoots for PCR at the end of Step 7 were picked only when they showed a positive result in GUS staining. The primers used in the PCRs were for testing the presence or absence of the *nptII* gene.

All surviving shoots at the end of Step 7 were transferred to rooting medium (Step 8). After the development of roots, the shoots were put into the soil and further cultured in the greenhouse to obtain T1 seeds. The seeds were germinated in petri-dishes with 3 layer filter papers wetted thoroughly and grew at 24 °C in darkness for 5 days, which gave etiolated seedlings. Seedlings of the next (T2) generation were tested again for PCR, gfp fluorescence and GUS staining. The same T2 seeds were later analyzed for the identification of homogeneous marker-free individuals by splitting seedlings, preparing cotyledonary node explants (CNEs; two per seedling), and regenerating/growing one CNE on medium supplemented with kanamycin and the other CNE on medium without. Figure 2 presents an example of the responses of CNE explants, also as an example for the responses that were observed in earlier tests. Subsequently, all explants with or without regenerants were stained for GUS.

## Additional Information

**How to cite this article**: Qi, W. *et al.* Screening for recombinants of *Crambe abyssynica* after transformation by the pMF1 marker-free vector based on chemical selection and meristematic regeneration. *Sci. Rep.* **5**, 14033; doi: 10.1038/srep14033 (2015).

## Figures and Tables

**Table 1:** The scheme of DEX treatments and 5-FC selection to which crambe GM lines were subjected for the generation of recombinant plants.

	Step 1	Step 2	Step 3	Step 4	Step 5	Step 6	Step 7	Step 8
Time	6 weeks	2 weeks	2 Weeks	3 weeks	3 weeks	3 weeks	3 weeks	6 weeks
Medium	RT	RG	RG	RG	RG	RG	RG	RT
Agent	DEX	DEX	DEX + 5FC	5FC/Km	5FC	5FC	5FC	None
Explants	Shoot	Axillary buds		Shoots	Shoots	Shoots	Shoots	Shoots
Analysis						PCR + GUS	PCR + GUS	

Note: RT = Rooting, RG = Regeneration. The regenerating shoots from the axillary buds were divided in Step 4, and part of them was subjected to 5-FC selection; and the rest to Km selection. In the other steps, only 5-FC was used for selection. At the end of Step 6 and Step 7, all of the surviving regenerating shoots were checked by GUS-staining, part of them was also checked by PCR.

**Table 2:** The number of crambe shoots and axillary buds subjected to the different DEX treatments for the two chosen T0 lines.

T0 line	DEX Con. (μM)	Shoots on rooting medium	Axillary buds on regeneration medium
**1**	0	16	62
	5	18	60
	15	19	62
	25	13	28
**Total**		66	212
**2**	0	8	23
	5	9	19
	15	16	40
	25	13	26
**Total**		46	108

**Table 3:** The numbers of surviving crambe GM shoot-clusters at the end of each step for the generation of recombinant plants.

Line	# No. of plants put on rooting medium (Step 1)	# No. of axillary buds isolated (Step2)	# No. of axillary buds put on 5-FC (Step 3)	# No. of axillary buds at the start of Step 4	End of Step 4	End of Step 5	End of Step 6	End of Step 7*	GUS + after Step 7
1	66	212	212	201	346	57	32	18 (4;5)	4
2	46	108	108	101	141	12	26	15 (4;4)	14

Note: As shown in the table, from Step 3 to the start of Step 4, a few axillary bud explants were discarded because that they gave no green regeneration shoots.

^*^Between brackets are the number of axillary buds at step 2 from which the surviving shoot cluster are derived and the number of original rooting plants at step 1, from which they are derived respectively.

**Table 4:** GUS staining and PCR tests on T1 crambeseedling obtained from the recombinant shoots identified earlier.

Seedlings	GUS staining		PCR			
	Blue	White	*gfp*		*nptII*	
			Negative	Positive	Negative	Positive
T1Family1	19	1	20	0	20	0
T1Family2	18	2	20	0	20	0
Line 2 (Without treatment)	18	2	1*	19	1*	19

Note: Two T1 families (T1 Family 1 and T1 Family 2) from marker-free shoots that were confirmed twice by PCR to be marker-free (shown in Figure 7) were selected. T1 seedlings from the Line 2 shoot without DEX treatment and 5-FC selection were used as control. From each of the families, 20 seedlings were prepared for GUS staining and PCR testing respectively. The asterisks indicate that the results were on the same individual seedling.
