# Erratum: Screening for recombinants of *Crambe abyssinica* after transformation by the pMF1 marker-free vector based on chemical selection and meristematic regeneration

**DOI:** 10.1038/srep15306

**Published:** 2015-10-20

**Authors:** Weicong Qi, Iris E. M. Tinnenbroek-Capel, Elma M. J. Salentijn, Jan G. Schaart, Jihua Cheng, Christel Denneboom, Zhao Zhang, Xiaolin Zhang, Han Zhao, Richard G. F. Visser, Bangquan Huang, Eibertus N. Van Loo, Frans A. Krens

Scientific Reports 5: Article number: 14033; 10.1038/srep14033 published online: 09112015; updated: 10202015.

The original version of this Article contained an error in the title of the paper, where the word “*abyssinica*” was incorrectly given as “*abyssynica*”.

In addition, the Acknowledgements section contained typographical errors.

“This study was funded by the European Union 7th Frame Work Project ‘Industrial Crops producing added value Oil for Novel chemicals’ (ICON, FP7-KBBE, 211400); and Agriculture Science and Technology Innovation Fund (CX(11)4034), Jiangsu Key Laboratory for Bioresources of Saline Soils (JKLBS2012004).”

now reads:

“This study was funded by the European Union 7th Frame Work Project ‘Industrial Crops producing added value Oil for Novel chemicals’ (ICON, FP7-KBBE, 211400); and Jiangsu Agriculture Science and Technology Innovation Fund [cx(12)2032], Jiangsu Key Laboratory for Bioresources of Saline Soils (JKLBS2012004).”

These errors have now been corrected in the PDF and HTML versions of the Article.

